# Establishment of a hospital-based health care workers surveillance programme to keep them safe during the COVID-19 pandemic

**DOI:** 10.7189/jogh.10.0203100

**Published:** 2020-12

**Authors:** Diane Woei-Quan Chong, Vivek Jason Jayaraj, Sanjay Rampal, Mas Ayu Said, Nik Daliana Nik Farid, Rafdzah Ahmad Zaki, Noran Naqiah Hairi, Victor Chee-Wai Hoe, Marzuki Isahak, Sasheela Ponnampalavanar, Sharifah Faridah Syed Omar, I-Ching Sam, Nazirah Hasnan, Hang-Cheng Ong, Adeeba Kamarulzaman, Chiu-Wan Ng

**Affiliations:** 1 Centre for Epidemiology and Evidence-based Practice, Department of Social and Preventive Medicine, Faculty of Medicine, University of Malaya, Kuala Lumpur, Malaysia; 2 Ministry of Health Malaysia, Putrajaya, Malaysia; 3 Department of Medicine, Faculty of Medicine, University of Malaya, Kuala Lumpur, Malaysia; 4 Department of Medical Microbiology, Faculty of Medicine, University of Malaya, Kuala Lumpur, Malaysia; 5 Department of Rehabilitation Medicine, Faculty of Medicine, University of Malaya, Kuala Lumpur, Malaysia

Severe acute respiratory syndrome coronavirus 2 (SARS-CoV-2) is a highly transmissible virus, making hospitals potential loci for outbreaks and placing health care workers (HCWs) at risk of acquiring the infection. The high demand for hospital care and the associated morbidity and mortality in the early phases of the coronavirus disease 2019 (COVID-19) pandemic has highlighted the urgent need to protect the safety and health of HCWs. With over 15.7 million cases [1], countries worldwide are currently in different stages of COVID-19 transmission. Countries are gradually re-opening their economies and easing large-scale restrictive public health measures. However, there remains clusters of cases in high vulnerability locations such as in the health care setting [2]. Therefore, it remains salient to protect the hospital community from being a COVID-19 transmission hub. In April, 22073 cases of COVID-19 among HCWs from 52 countries were reported to the World Health Organization [3]. In June, it was estimated that 7% of all infections worldwide were among HCWs [4]. As of July, Italy, a nation particularly affected by the epidemic, had alone reported more than 29800 infections among HCWs, amounting to 12% of all infections [5], further crippling a health care system under duress.

In managing patients with COVID-19 infection, proper use of personal protective equipment is crucial for the protection of HCWs [6]. However, HCWs may also acquire infection from other HCWs or the community. Thus, a comprehensive hospital HCW surveillance programme, taking into consideration all these sources of infection, is essential to protect HCWs, their families, and by extension, their patients. This programme will need to detect those at risk of infection at an early stage to allow for appropriate quarantine measures to limit risks of transmission. Here, in this viewpoint, we describe one such programme implemented at the University of Malaya Medical Centre (UMMC), a 1600 bedded tertiary teaching hospital with more than 5600 HCWs, located in Kuala Lumpur, Malaysia.

**Figure:**
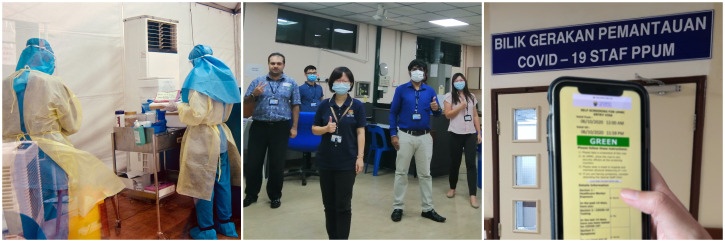
Photo: Left panel: Healthcare workers in personal protective equipment. Middle panel: The team operating the hospital’s COVID-19 Preparedness and Response Centre. Right panel: The University Malaya Medical Centre’s Entry Visa- a COVID-19 surveillance tool for health care workers, patients and visitors (Photos from Medical Development and Health Sciences Division, Department of Professional Development and Medical Development, UMMC and Xiong Cailian’s collection, used with permission).

Malaysia reported its first case of COVID-19 on January 24, 2020 [7]. Subsequently, several public hospitals in Malaysia, including UMMC, are designated as COVID-19 hospitals, allowing these facilities to focus on the care and management of COVID-19 patients. It is the current policy to isolate and treat all confirmed COVID-19 patients in these designated hospitals [8]. In January, UMMC created a multi-disciplinary committee, chaired by a senior infectious disease specialist, to provide leadership and to formulate, and implement policies for the hospital. The resultant hospital preparedness plan includes multiprong strategies for managing patients as well as to identify and mitigate the risk of disease transmission within the hospital. The HCW surveillance programme, led by a public health physician, is an integral component of this plan. It consists of five main sections: case notification, contact tracing, risk assessment, daily symptom surveillance, and outbreak management.

One of the main pillars of the Malaysian pandemic preparedness programme is the public health approach of contact tracing complemented with targeted testing of close contacts. From the outset of the pandemic, the Ministry of Health (MoH) Malaysia through its district health offices has been implementing intensive contact tracing for every confirmed COVID-19 case nationwide, and testing of all identified contacts. Within the confines of the hospital population, UMMC took over the responsibility for contact tracing and testing activities among its HCWs. In general, these activities follow the criteria and guidelines issued by the MoH but are adapted to allow for the nature of clinical work performed by HCWs.

The HCW surveillance team is notified when any patient or HCW tests positive. Listings of close contacts are obtained through interviews with the index case and supplemented with information obtained from the hospital’s electronic medical records (EMR). The EMR enables tracking of patients’ movements throughout their care processes within the hospital and the identification of HCWs who may have interacted with them during their periods of infectivity. Subsequently, all identified close contacts will have their risk of exposure evaluated and stratified into high, medium or low risk. It includes a list of standardised questions such as the duration of exposure, clinical symptoms of the COVID-19 case and whether the case was wearing a face mask and whether an aerosol-generating procedure was performed as well as the type of personal protective equipment that was used by the HCW. Risk assessment is being carried out for every new exposure to a COVID-19 case. These HCWs are tested with real-time PCR for SARS CoV-2. A 14-day and 7-day quarantine notice is issued to HCWs with high and medium risk of exposure, respectively. Meanwhile, a two-day sick leave is provided for HCWs with low risk of exposure while awaiting PCR results, which are available within 24 hours. The management algorithm for HCWs with work-related exposure [9] is adopted and adapted from MoH’s guidelines [8] for UMMC’s specific needs and is continuously being updated based on new evidence.

All HCWs, with identifiable risk, are then placed under daily symptom surveillance for 14 days from the last exposure to a COVID-19 case. HCWs were initially contacted via social messaging systems and direct calls to ensure compliance to this daily surveillance. An online risk assessment and symptom surveillance system, linked to the EMRs and human resource records, was subsequently developed and deployed to automate the process. HCWs now receive an automated reminder through mass texting service as a prompt for self-assessment and self-reporting of symptoms through the UMMC staff portal. To ensure ethical compliance, all the records of the risk assessment and symptom surveillance are treated as medical records and included in the EMR.

The outbreak management team continuously monitors all activities to detect any secondary propagation of disease transmission within the hospital community. An epidemiologist leads a multidisciplinary outbreak investigation into all potential hospital clusters to provide remedial plans for infection control and prevention activities. In addition to these core components of disease surveillance, UMMC has also instituted a daily COVID-19 symptom roll call for all HCWs, which is incorporated into their daily attendance records. HCWs with symptoms will be provided with appropriate advice and care, and if appropriate, referred to a staff health clinic for assessment.

Between February and July 2020, a total of 2401 risk assessments were carried out among 1408 HCWs who were either exposed to a COVID-19 case in the hospital or the community. Of these assessments, 16.5% were stratified as moderate or high-risk exposure. In extension, 1292 HCWs underwent at least 14 days of symptom surveillance. Cumulative incidence of COVID-19 amongst health care workers was 0.3%. The number of HCWs who underwent risk assessment and were placed under active daily symptom surveillance is subject to changes due to the dynamic situation of COVID-19 pandemic in the country.

The rapid spread of the COVID-19 pandemic necessitates that every hospital takes extraordinary measures to protect its health workforce. We believe that the HCW surveillance programme implemented in UMMC limits intrahospital transmission of COVID-19 and ensures better patient and staff safety during these difficult times.
